# Assessment of Positive Cardiac Remodeling in Hypertrophic Obstructive Cardiomyopathy Using an Artificial Intelligence–Based Electrocardiographic Platform in Patients Treated With Mavacamten

**DOI:** 10.1016/j.mcpdig.2024.04.002

**Published:** 2024-04-10

**Authors:** Mustafa Suppah, Kaitlin Roehl, Kathryn Lew, Reza Arsanjani, Steven Lester, Steve Ommen, Jeffrey Geske, Konstantinos C. Siontis, Hartzell Schaff, Said Alsidawi

**Affiliations:** a Department of Cardiovascular Diseases, Mayo Clinic, Scottsdale, AZ; b Department of Cardiovascular Medicine, Mayo Clinic, Rochester, MN; c Department of Cardiac Surgery, Mayo Clinic, Rochester, MN

Hypertrophic cardiomyopathy (HCM) is a common inherited heart condition characterized by abnormal thickening of the heart muscle, broadly categorized into obstructive hypertrophic cardiomyopathy (oHCM) and nonobstructive hypertrophic cardiomyopathy types. oHCM, accounting for 70% of cases, is identified by left ventricular outflow tract (LVOT) obstruction with a gradient of ≥30 mm Hg at rest or with provocation.^1^^,^^2^

Although surgical myectomy is considered the traditional treatment for patients with symptomatic oHCM, recent advancements have introduced pharmacologic alternatives. Notably, mavacamten, a novel cardiac myosin inhibitor, has demonstrated promising results in improving symptoms of individuals with oHCM.^3^^,^^4^ Whether mysoin inhibitors result in positive cardiac remodeling, improve phenotype severity, or reduce the burden of atrial arrythmias is still unknown.

This study explores potential cardiac remodeling using an artificial intelligence–based electrocardiographic platform (AI-ECG) in patients with oHCM and treated with mavacamten.

## Methods

Sixteen patients with consecutive oHCM (average age, 64 years; 56% men) who completed 12-week treatment with mavacamten were analyzed. Patients with known atrial fibrillation (AF), postoperative AF, or wide complex QRS (left bundle-branch block and paced rhythm) were excluded.

The primary outcomes included estimated probabilities of AF, low ejection fraction (EF) (<35%), and HCM phenotype severity as assessed by validated ECG-based AI algorithms.^5^

The algorithm used was internally developed and heavily validated with an area under curve (AUC) of 0.96 (5). Thousands of electrocardiograms from patients with confirmed HCM as well as thousands of controls were fed to a convolutional neural network. The network was then tested using the internal validation dataset. Electrocardiogram segments that are believed to most influence the algorithm were atrial depolarization segment, QRS complex voltage and fragmentation, and ST-T segment abnormalities. However, when saliency maps were applied, we found that the ST-T segment had the greatest impact on the algorithm rather than the QRS voltage.^6^ Subsequent external evaluations suggested that the AI probability for HCM declines in accordance with hemodynamic measures (LVOT gradient) and circulating biomarkers (NTproBNP) of treatment response, which we used as a surrogate for phenotype severity in this article.^7^

The patients’ standard 12-lead ECGs pretreatment and after 12 weeks of treatment were analyzed with these algorithms. We also evaluated changes in LVOT gradient, LA volume, and transverse LA diameter.

Continuous variables were reported as mean ± SD for normally distributed variables and median with interquartile range for nonnormally distributed variables. Categorical variables were reported as frequency with percentage.

## Results

### ECG-AI Outcomes

Patients treated with mavacamten demonstrated a significant reduction in ECG-AI-predicted AF risk, from a median of 14.14% (IQR, 22.31) to a median of 9.66% (IQR, 20.42; *P*=.020) (Table). Mavacamten-treated patients also showed a significant decrease in HCM phenotype severity from 71.65% (IQR, 79.78) to 6.76% (IQR, 86.04; *P*=.001). Finally, there was a nonsignificant change in AI-ECG determined reduced EF probability, from 0.7% (IQR, 3.68) to 1.15% (IQR, 4.00; *P*=.77). When ECGs before and after treatment were directly compared, indeed the most striking changes were improvement in ST-T segments, likely owing to the improvement in LVOT obstruction (Figure).

**Table:** Comparison of Outcomes of ECG-AI, Clinical, and Echocardiographic Data Before and 12 Weeks After Mavacamten Therapy

Variable	Baseline	12 wk after mavacamten	*P*
Probability of AF (%)	14.14 (IQR, 22.31)	9.66±20.42	0.020^a^
HCM phenotype severity by AI-ECG (%)	71.65% (IQR, 79.78)	6.76% (IQR, 86.04)	0.001^a^
LVOT gradient (mm Hg)	72.50 (IQR, 20)	36.50 (IQR, 47.75)	0.005^a^
TLAD	46.81 4.79	44.08 5.22	0.001^b^
LA volume index (mL/m^2^)	42.12±12.40	37.93±12.78	0.07^b^
Probability of EF <35% (%)	0.70 (IQR, 3.68)	1.15 (IQR, 4.00)	0.78^a^

AF, atrial fibrillation; AI, artificial intelligence; ECG, electrocardiogram; EF, ejection fraction; HCM, hypertrophic cardiomyopathy; LA, left atrial; LVOT, left ventricular outflow tract.

a Wilcoxon signed rank test.

b Paired *T* test.

**Figure:**
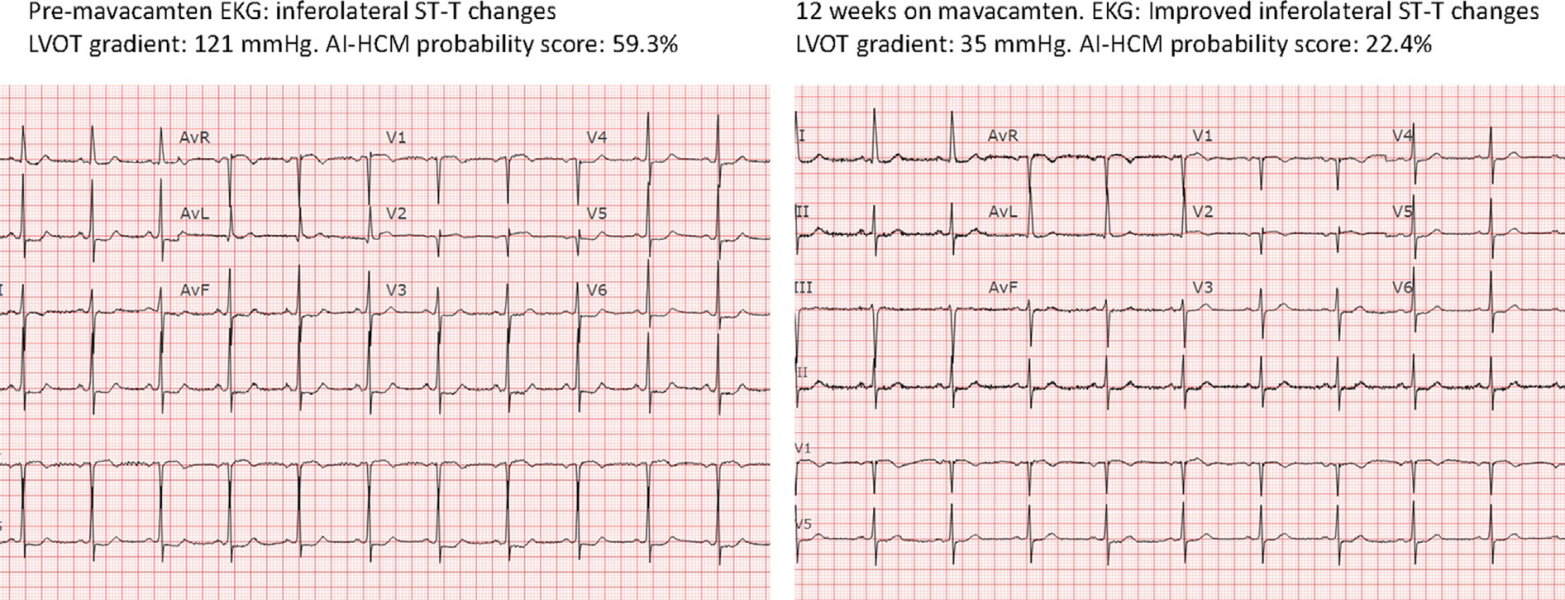
A case example of a patient’s electrocardiogram done before and after 12 weeks of treatment with mavacamten showing improvement in ST-T segment with the improvement in left ventricular outflow tract (LVOT) gradient, correlating with a drop in ECG-AI Hypertrophic cardiomyopathy (HCM) probability score.

### Other Clinical and Echocardiographic Outcomes

Mavacamten-treated patients showed a significant improvement in LVOT gradient from a median of 72.5 mm Hg (IQR, 20 mm Hg) at baseline to 36.5 mm Hg (IQR, 47.8 mm Hg; *P*=.005) after 12 weeks of therapy (Table).

Mavacamten therapy was associated with a reduction in the transverse LA diameter from 46.81± 4.79 to 44.08± 5.22 (*P*=.001). There was a nonstatistically significant trend in reduction of LA volume index from 42.12± 12.40 to 37.93±12.78 (*P*=.07).

## Discussion

This study investigated cardiac remodeling in patients with oHCM and treated with mavacamten using AI-ECG. Key findings include a significant reduction in AI-ECG determined AF risk associated with 12 weeks of mavacamten treatment. Furthermore, mavacamten utilization was associated with a significant improvement in the HCM phenotype severity as assessed by AI-ECG ability to detect HCM, despite some residual LVOT gradient at the time of analysis (12 weeks, before allowing dose up-titration), suggesting a potential broader impact on cardiac remodeling beyond relieving LVOT obstruction. No significant changes in the AI-ECG probability of developing low EF were observed. Clinically, there was a significant reduction in LVOT consistent with previous reports.^3^^,^^4^

## Conclusion

This study highlighted mavacamten’s potential for positive cardiac remodeling as assessed by AI-ECG. Although these results are intriguing, the analysis is limited by sample size; hence, larger and long-term studies are needed to explore myosin inhibitors’ sustained effects on positive cardiac remodeling in oHCM.

## Potential Competing Interests

The authors report no competing interests.

## Ethics Statement

The Mayo Clinic Institutional Review Board(s) approved the study and determined that due to the retrospective nature and low risk of the study, patient consent was deemed unnecessary. However, all patients provided consents for their data to be used for research purposes when they registered as a patient at Mayo Clinic.
